# A Fault Diagnosis System for a Pipeline Robot Based on Sound Signal Recognition

**DOI:** 10.3390/s22093275

**Published:** 2022-04-24

**Authors:** Hai Cao, Jinpeng Yu, Yu Wang, Liang Zhang, Jongwon Kim

**Affiliations:** 1 School of Mechanical, Electrical and Information Engineering, Shandong University, Weihai 264200, China; caohai@sdu.edu.cn (H.C.); 202037543@mail.sdu.edu.cn (J.Y.); 201900800394@mail.sdu.edu.cn (Y.W.); zhangliang@email.sdu.edu.cn (L.Z.); 2 Department of Electromechanical Convergence Engineering, Korea University of Technology and Education, Cheonan-si 31253, Korea

**Keywords:** fault diagnosis system, sound sensor, audio classification, pretrained model, transfer learning

## Abstract

Timely and accurate identification of fault types at the early stage of minor faults is significant for cutting off fault evolution. In order to have a clear understanding of the pipeline robot’s own situation in the pipeline, this paper proposes a fault diagnosis system for pipeline robots based on sound signal recognition. This can effectively reduce the probability of serious faults such as shutdown and loss of control in the pipeline without affecting the safe operation of the pipeline robot, which is a key issue to improve the reliability of the pipeline robot. The system consists of a combination of three parts: hardware, software, and algorithm. On the one hand, Raspberry Pi is the core module, while on the other hand, it is also responsible for the data transmission between the various modules, including storing the original sound signals collected by the sensors and transmitting the diagnosis results to the upper computer software interface. The proposed system is validated on the dataset collected by the data experimentation platform. The experimental results show that the proposed fault prediction method obtains advanced results on this dataset, verifying the effectiveness and stability of the proposed fault diagnosis system for pipeline robots based on sound signal recognition.

## 1. Introduction

Machine fault diagnosis is to monitor the mechanical equipment and identify the state when the fault occurs. To identify and classify faults, multiple sensors are installed to collect data, such as sound data or vibration data, which are then identified to determine if a fault has occurred [1].

Pipeline robots are used for inspection work of large LPG pipelines, aiming to achieve efficient inspection of LPG pipelines and prevent safety accidents from occurring. Pipeline robots can enter the interior of pipelines with relatively small spaces and complete pipeline inspection and maintenance work instead of workers, which can greatly improve the efficiency of pipeline maintenance operations and reduce maintenance costs, which is an inevitable demand for industry development. In the pipeline robot system, electronic components, drive parts and sensors are subject to environmental factors such as temperature and humidity and other factors such as looseness and poor contact, which inevitably produce aging and other problems. How to timely and accurately identify the type, size, and location of faults at the early stage of minor faults is of great significance to cut off the evolution and propagation of faults. The study of accurate fault self-diagnosis methods for pipeline robots can effectively reduce the probability of serious faults such as robot downtime and loss of control in the pipeline and avoid the situation that the robot cannot exit the pipeline due to serious faults, which is a key issue to improve robot reliability.

Fault diagnosis is an important way to ensure the reliability and safety of industrial systems and reduce the risk of accidental failure [2]. Early fault diagnosis can optimize maintenance schedules while maximizing machine utilization and avoiding catastrophic damage [3]. Acoustic signals are an important source of information reflecting the operating status of equipment, and acoustic signals have the advantages of easy acquisition, non-contact measurement, and low cost [4]. Sound signal analysis has become an effective method for monitoring. At first, the condition monitoring of the machine relied on the experience of skilled technicians, but the decision-making process was highly subjective [5]. This depends on the operator’s experience and ability to correctly hear and perceive sounds [4], so more automated methods are needed, and automatic audio classification is of great significance in today’s era.

Traditional machine fault diagnosis includes three main stages: sensor signal acquisition, feature extraction and selection, and fault classification. Traditional feature extraction methods are based on a manual selection of features. If these manually selected features are not suitable for the task, the fault classification performance will be significantly reduced. Therefore, traditional fault diagnosis methods have certain limitations [1].

In recent years, the development of deep learning has provided opportunities for data-driven fault diagnosis and provided effective solutions for overcoming the above limitations [6]. Through model training, the deep model can automatically select useful features that accurately predict the subsequent classification stage based on the training data, and has been applied to the field of mechanical fault diagnosis. However, deep learning methods still have some problems. Deep architecture models have multiple hidden layers. As the number and size of hidden layers increase, training a very large network from scratch usually requires a lot of computing and time resources.

A reliable diagnostic model needs to rely on sufficient fault data, but in most cases, the machine is in normal operation. For the sake of safety, some machines are not allowed to continue running in a fault state [3], and it is difficult to collect enough fault data. Therefore, it is difficult to train a deep learning model with good generalization performance and high accuracy [7].

Transfer learning is a promising tool to address this problem [8,9,10]. Briefly, given a source domain Ds and a target domain Dt, transfer learning attempts to apply the knowledge previously learned from Ds to Dt. Transfer learning can help train the target model by transferring weights, keeping the weights of the starting layer of the model unchanged, and fine-tuning the higher layers of the neural network through the target dataset. It provides ideas to solve the lack of fault data and accelerate the training of neural networks.

Currently, the fault diagnosis system proposed in this paper is for an individual robot. Experiments were first performed on the pipeline robot mentioned in this paper, and the health of this robot can be monitored in real time. If the model is used to monitor the health of other robots, it also needs to be equipped with a Raspberry Pi and the algorithm. The next step is to implement a single centralized system to diagnose the faults of all robots.

The main contributions of this paper are as follows:

(1) We propose an online fault diagnosis system for pipeline robots based on sound signal recognition. By identifying the sound signals obtained by sensors, the working conditions of pipeline robots can be judged, and it can be used to perform predictive maintenance when needed to extend the life of pipeline robots. Without affecting the safe operation of the pipeline robot, the probability of serious failures such as shutdown and loss of control of the robot in the pipeline is reduced.
(2) Based on the idea of deep transfer learning, an end-to-end fault diagnosis method is proposed. First, the original sensor data are converted into time–frequency images through the short-time Fourier transform method, the underlying features are extracted by a pretraining network, and then the time–frequency images are used to fine-tune the higher levels of the neural network. The model solves the problem that small datasets cannot be trained in deep neural networks, and the use of transfer learning greatly reduces training time.

The rest of this paper is organized as follows: Section 2 starts with a brief review of related research. The fault diagnosis system is designed in Section 3. Experimental results of the proposed system are presented in Section 4. Lastly, conclusions and remarks on possible further research are given in Section 5.

## 2. Related Research

Data fault diagnosis is a typical fault diagnosis method that has received extensive research in recent years. Traditional fault diagnosis is based on the manual selection of features, and the performance of the classification task is degraded if the manually selected features are not suitable for the task. Machine learning techniques have been applied in data-driven fault detection based on, for example, support vector machines, artificial neural networks, and expert systems. Shatnawi and Al-Khassaweneh proposed an extended neural network for internal combustion engine fault diagnosis [11]. Yin and Hou investigated the advantages of SVM-based fault diagnosis and process monitoring [12]. Qin et al. used random forests for the classification of bearing fault features [13]. However, machine learning models cannot learn sufficiently rich fault information, which limits the accuracy of the final diagnosis to meet the requirements of modern fault diagnosis.

With the rapid development of deep learning, the above problems can be solved; unlike the shallow representation of machine learning, deep learning often has dozens of layers, and many deep learning models have been applied in fault diagnosis. Zhao et al. investigated the application of DL in machine health monitoring [14]. Cho et al. proposed the use of recurrent neural networks and dynamic Bayes to solve the fault detection of induction motors [15]. Sun et al. designed an automatic coding-based neural network for induction motor diagnosis to achieve accurate fault prediction [16]. A reliable diagnostic model relies on having sufficient fault data; however, it is difficult to collect sufficient fault data in most cases. Therefore, it is difficult to train a fault diagnosis model with good generalization performance and high accuracy.

With the rapid development of deep learning, transfer learning and sound signal analysis have been successfully applied in various intelligent fault diagnosis tasks [17,18,19]. Wen et al. designed a fault diagnosis method for induction motors based on sparse autoencoders. Experimental results show that the prediction accuracy of this method is greatly improved, compared with traditional algorithms [2]. Wen et al. [20] converted the vibration signal into an image and used it in an improved CNN model to obtain advanced classification accuracy. Kong et al. proposed an audio neural network (PANNS) trained on a large-scale AudioSet dataset and migrated this network model to other tasks. PANNs are very useful when fine-tuning based on a small quantity of data on a new task [21]. Chen et al. proposed a new migration learning method, which uses sound data obtained from the operating switches on-site to evaluate the structural state of the rails, and achieved accurate failure prediction. The system can issue an early warning of railway cracks and help railway operators to carry out maintenance work in time [22]. Shao et al. designed a high-precision machine fault diagnosis based on deep transfer learning and conducted experiments on the dataset of induction motors to verify the effectiveness and generalization of the method [1].

Based on the above inspiration, a fault diagnosis method based on fine-tuned ResNet50 model is proposed to solve the fault classification problem of pipeline robots.

## 3. Proposed Fault Diagnosis System

**Figure 1:**
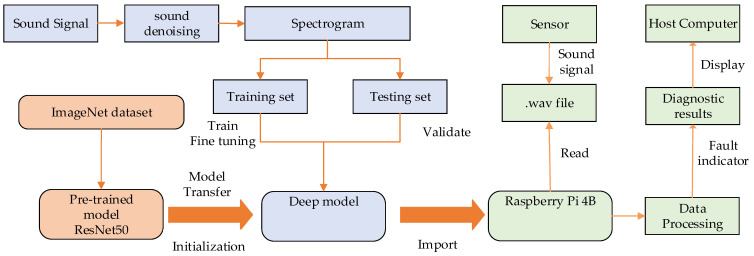
The framework of the fault diagnosis system.

The pipeline robot fault diagnosis system based on sound signal recognition consists of three parts: hardware, software, and algorithm. The hardware part includes the sound sensor and Raspberry Pi. The Raspberry Pi is the core module. On the one hand, it is responsible for installing the program running environment, denoising the collected sound by wavelet threshold denoising algorithm, preprocessing the signal, extracting features, and deploying the trained sound model. On the other hand, it is also responsible for the data transfer between the modules, including storing the raw sound data collected by the sensors and transferring the diagnosis results to the host computer interface. The algorithm part proposes an end-to-end fault prediction method. ResNet50 was selected as the pretraining model of a deep convolutional neural network, and the fault classification problem of the pipeline robot was solved by fine-tuning the ResNet50 model based on the idea of migration learning. The underlying features were extracted using the pretrained network and the higher levels of the neural network were fine-tuned using time–frequency images. The fault diagnosis system of the pipeline robot based on sound signal recognition is shown in Figure 1.

### 3.1. Hardware

#### 3.1.1. Pipeline Robot

**Figure 2:**
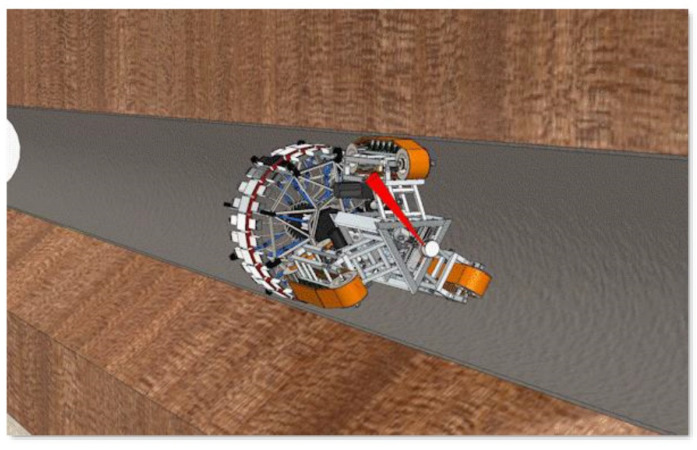
The operation of the pipeline robot in the pipeline (See Supplementary Material for dynamic version).

The pipeline robot [23] can enter the pipeline in a relatively narrow space and replace the workers to complete the pipeline inspection and maintenance work, which can greatly improve the efficiency of pipeline maintenance and reduce the cost of maintenance. The pipeline robot is accompanied by various sounds during the internal operation of the pipeline. The research goal of fault diagnosis is to be able to quickly detect some common problems of the pipeline robot. The operation of the pipeline robot in the pipeline is shown in Figure 2.

#### 3.1.2. Raspberry Pi and Sound Sensors

The identification module is the core of the entire system. Affected by the external operating environment, the equipment itself should also meet the requirements of small size and easy installation. Based on the above considerations, a high-performance embedded motherboard Raspberry Pi 4B was used, loaded with the Raspbian operating system.

On the one hand, the Raspberry Pi needs to deploy a failure prediction algorithm. On the other hand, it is also responsible for the data transmission between the various modules, including storing the original sound data from the sensors and transmitting the diagnosis results to the upper computer software interface.

**Figure 3:**
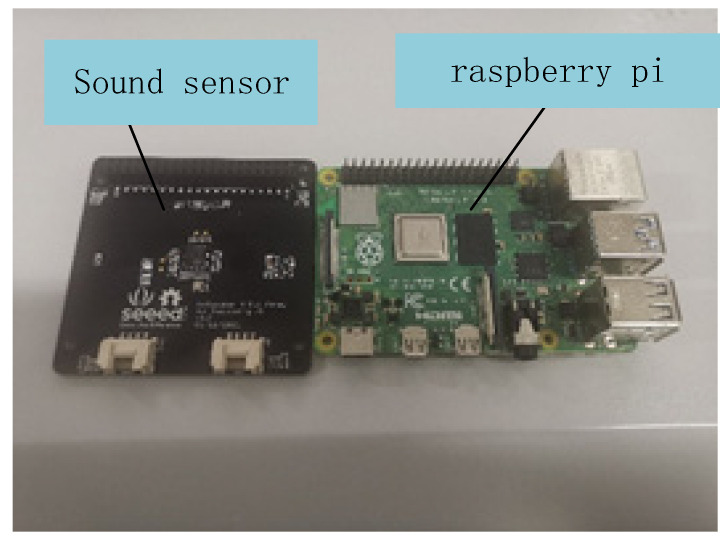
Raspberry Pi and sound sensor used for sound acquisition.

The sound acquisition module is the input of the entire system. In terms of hardware selection, the sound sensor uses a microphone array that can enhance speech and suppress noise from interference detection, reducing the noise interference of the sound signal. The microphone array can also be used in combination with the Raspberry Pi, which is more convenient for the transmission of sound data. Raspberry Pi and sound sensor are shown in Figure 3.

### 3.2. Software

**Figure 4:**
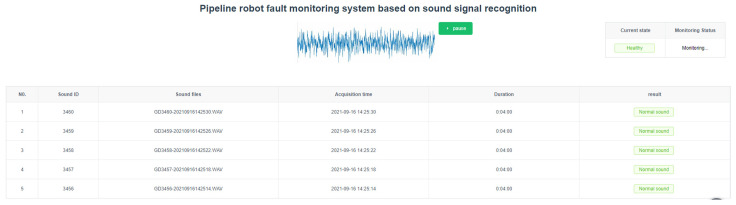
Sound monitoring information page.

The health status is a monotonic function that changes over time, and it will eventually cross a threshold, leading to a fault status. Through the real-time changes in the host computer interface data, one can have a better understanding of the health status of the pipeline robot. The upper computer interface is shown in Figure 4. The host computer interface can display the current operating status and changes in the sound signal and view the past monitoring conditions.

### 3.3. Fault Diagnosis Method Based on Deep Transfer Learning

#### 3.3.1. Data Preprocessing

When the pipeline robot is operating, the collected sound signals are not pure and are mixed with noise to a greater or lesser extent. The presence of noise has an impact on the fault diagnosis based on the sound signal. Through the analysis of the sound signal collected in the field, it was found that the signal-to-noise ratio is low. By analyzing the advantages and disadvantages of the sound signal time–frequency domain analysis methods, it was found that the wavelet basis function can modulate its vanishing moment to make the high-frequency noise component in the sound signal decay quickly, and its symmetry, tight branching, and orthogonality can be used to better detect the transient sound signal, so the wavelet threshold denoising method was used to denoise the sound signal.

The acquisition frequency was 48 kHz, the number of sampling points was 4096, the number of decomposition layers was selected as 3, the wavelet basis was selected as dB 6, the threshold value was uniformly selected as a fixed threshold, and the threshold function was selected as a soft threshold function. Table 1 shows the signal-to-noise ratios of the four types of sound signals before and after using the wavelet threshold denoising algorithm. It can be seen that the signal-to-noise ratio of the sound after denoising is higher, and the noise filtering effect is good. With this denoising method, the collected sound signal is enhanced, which is beneficial to obtaining reliable fault monitoring.

It is difficult to distinguish the corresponding fault types based on the original sound signal, but the difference between the time–frequency images can distinguish each type of fault, and it is suitable to further input these time–frequency images into CNN for feature extraction. First, the original sound signal was converted into a time–frequency image through the short-time Fourier transform method, which was used as the input of the following neural network. A spectrogram is a very common time–frequency analysis method and a useful tool for analyzing sensor signals for fault diagnosis [24,25]; in this study, the spectrogram was further converted into RGB images.

The image size was adjusted to 224×224, and the processed dataset was divided into training and testing sets. The training set was used to train the pretrained model and fine-tune its weights, while the testing dataset was used to verify the performance of the fine-tuned model and was not involved in the training process.

#### 3.3.2. Pretraining Model Construction and Fine-Tuning

Deep convolutional neural networks can automatically learn image features from input images, where the features learned at higher layers are more abstract than those learned at lower layers, and these abstract features help accurate classification. Generally, CNN contains three layers—namely, the convolutional layer, pooling layer, and fully connected layer—and several such layers are stacked to build a deep learning model, using the fully connected layer as the last layer to perform classification or regression.

The lower convolutional layers extract low-level features, such as edges and curves, which are suitable for common image classification tasks, while the operations in the subsequent layers can learn more abstract representations for different application domains. Thus, the weights of the starting layer can be migrated, and only the higher-level weights need to be learned from the new dataset; the process of updating the weights of the later layers is called fine-tuning. Its success depends in part on the distance between the source and target datasets. For similar datasets, it is possible to adjust only the fully connected layers, while for datasets with differences, the starting convolutional layer needs to be frozen, and the higher convolutional layer needs to be updated. This approach is faster than training from scratch because it essentially reduces the number of parameters that need to be trained.

**Figure 5:**
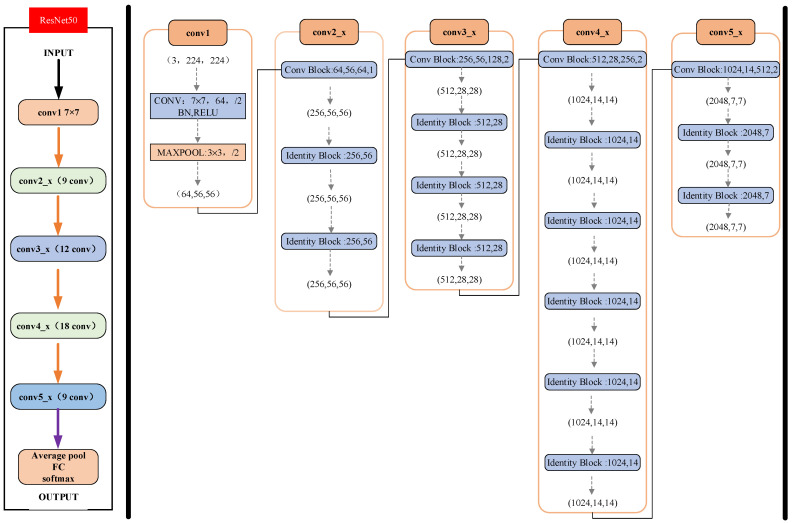
Detailed configuration of ResNet50.

The pretrained model used in this study was a 50-layer deep convolutional network created by Resnet50 [26], which achieved accurate classification performance on the ImageNet dataset. ResNet-50 is one of the most advanced network architectures for CNNs. By introducing residual learning, ResNet-50 can effectively avoid the gradient disappearance and degradation caused by deepening the layers of the network. Instead of simply learning the potential mapping between input x and output H(x) directly, ResNet-50 learns F(x) + x by adding the residual to the input through the residual between them, F(x) = H(x) − x. Resnet-50 has two basic blocks, named Conv Block and Identity Block; the details of this architecture model used in our study are shown in Figure 5. It consisted of several stacked Conv Blocks and Identity Blocks, with 49 convolutional layers and one SoftMax layer. ResNet-50 has superior performance in processing image classification tasks.

The ResNet50 model was trained on ImageNet. As the time–frequency image of the fault data is different from the natural image, it is necessary to fine-tune more residual blocks of the pretraining model. We transferred the weights of the first four residual blocks and fine-tuned the weights of the last residual block and the fully connected layer.

#### 3.3.3. Model Training

**Figure 6:**
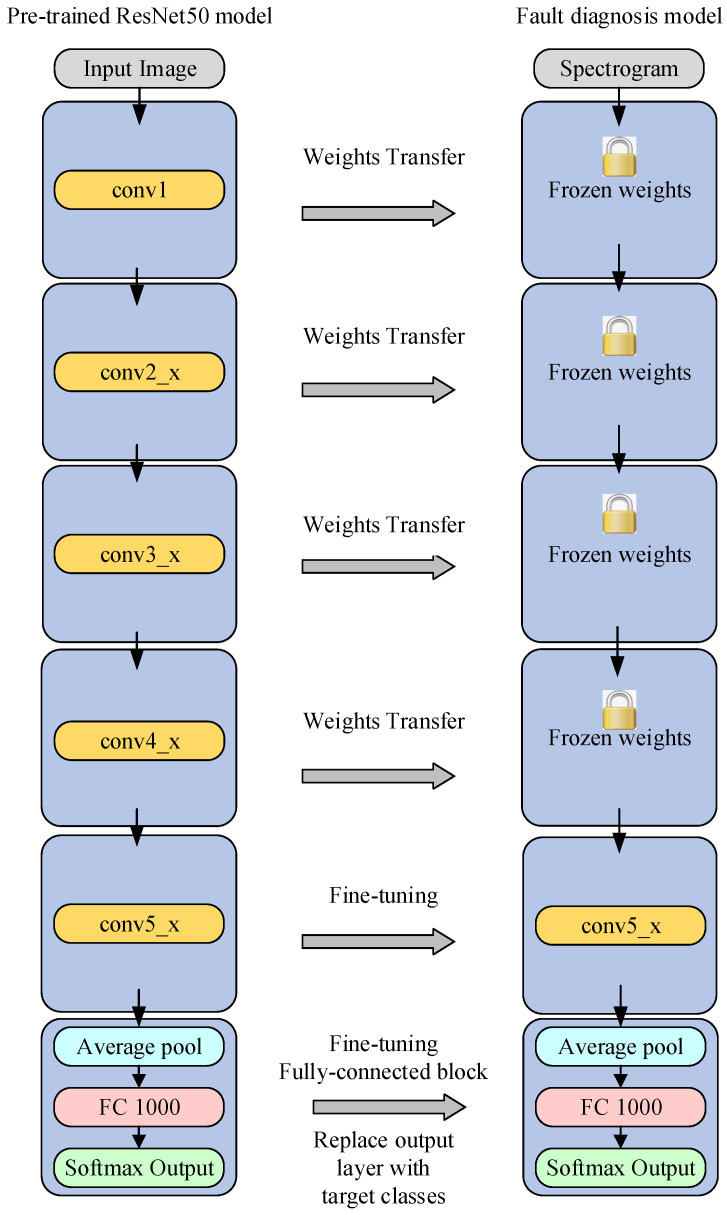
Transfer learning by using fine-tuning.

We adjusted the pretrained CNN by removing the output layer from the pretrained architectural model. A new output layer was added, the size of the output layer was determined by the number of pipeline robot working conditions, and the weights of the newly added output layer were initialized randomly. The last residual block and the fully connected layer were set as trainable, and during the training process, the starting layer was frozen, while the weights of the trainable layer were updated to minimize the error between the predicted and true labels, as shown in Figure 6. After a sufficient number of cycles, the fine-tuned deep learning model was saved.

A test dataset was used to verify the recognition performance of the model, and the fine-tuned model was used to diagnose the working status of the pipeline robot.

## 4. Experimental Verification

Python was used as the programming language to import the Pyaudio library to interact with the sound capture device for audio data, set the recording parameters, open the recording, and set the location to save the audio files. The database module was selected as the open-source MySQL database, and the data were interacted with the embedded module by calling the interface in the Pymysql library. The data table in the database stores five types of data—namely, ID number, detection time information, detection event results, and spectrogram feature.

**Figure 7:**
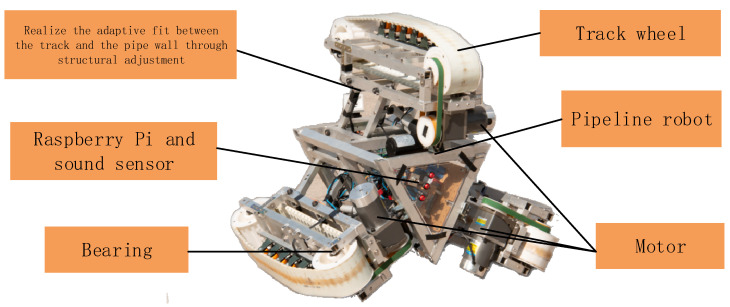
Integration of pipeline robot and fault diagnosis system.

As shown in Figure 7, the fault diagnosis experimental platform consists of a sound sensor, Raspberry Pi, database module, and upper computer. The sound signal sampling frequency was set to 48 kHz, the collected sound signal experiment set was divided into six groups, normal signals, and five kinds of fault signals; the length of each group of collected sound signals was 300 s, and each segment was 300 ms. The experimental environment was 64-bit Windows 10 operating system, CPU is Intel(R) Core(TM) i5-6500 @ 3.20 Hz, a graphics card is NVIDIA Ge Force GT 710, and memory is 8.00 GB.

We remotely logged in Raspberry Pi, which is powered by the built-in battery of the pipeline robot, and installed suitable environments—namely, python3.7 and pytorch1.8.1. With the proper record parameters, voice data were collected through the sensors in wav files. Then, python scripts would preprocess the wav files and extract the voice features by means of libsora library and python_speech_features package. Ultimately, we deployed the recognition model in Raspberry Pi, using the fault prediction algorithm to recognize sound signals. We embedded the fault diagnosis platforms in the pipeline robots, which implement real-time monitoring, storing the diagnostic result in databases and transferring that result to the upper computer interface to display.

To test the performance and verify the effectiveness of this fault diagnosis system, the proposed diagnosis system was tested on the sound dataset of a pipeline robot and compared with other deep learning methods. Finally, the operation of the whole system was observed.

### 4.1. Dataset and Experimental Setup

The dataset was collected from the experimental platform provided by the laboratory, in which six different operating conditions during the operation of the pipeline robot were simulated, and the sound data of the pipeline robot were collected by the sound sensor. The data included normal conditions and five fault conditions; the description of each working condition is shown in Table 2.

Each of these working conditions was considered a separate category. First, the audio files were framed and windowed, and the sound signals were converted into spectrograms by short-time Fourier transform and then further converted into RGB images. The whole dataset was divided into a training set and a testing set; each working condition contained 1000 training samples and 100 test samples. Therefore, the size of the training set was 6000, and the size of the testing set was 600.

The fault diagnosis of the pipeline robot can be regarded as a six-category task. The output layer of the ResNet50 model is replaced with a new layer, which contains six neurons, corresponding to six different working states, and performs random initialization of the weights. In order to fine-tune the model, we froze the first four residual blocks and updated the weights of the last residual block and the fully connected layer. In the training process, we used an Adam optimizer, the learning rate was set to 0.0001, and the batch size was set to 32. After fine-tuning the model, the test dataset was used to evaluate the model classification performance.

### 4.2. Results and Discussion

We used the Keras library for model training and fine-tuning. The performance of the model was evaluated by calculating the classification accuracy, and the experimental results are shown in Table 3. They were also compared with the fine-tuned VGG16 depth migration method and with the method of training the network model from scratch. To investigate the effect of the number of fine-tuning layers on the performance of the proposed model, different depth migration models were constructed for different fine-tuning layers [10], comparing the freezing of the first four residual blocks and the freezing of the first five residual blocks.

The results show that the method can accurately reflect the working state of the pipeline robot, outperforming other methods on this dataset, and obtains a high classification accuracy. Compared with VGG16, the deep transfer method (ResNet50 model) proposed in this paper has better classification accuracy, indicating that the method has better generalization ability. CNNs trained from scratch need trial-and-error to obtain the best performance, which takes much time, while the pretrained model proposed in this paper obtains the best hyperparameters that can be migrated to the fault classification task. Regarding the selection of fine-tuning layers, the results show that increasing the number of fine-tuning layers can overcome the source and target domains within a certain range and improve the accuracy.

System operation is stable during the monitoring period, proving the effectiveness and stability of the system for monitoring the health condition of the pipeline robot and further verifying its ability to solve engineering problems.

## 5. Conclusions and Future Research

In this paper, an online fault diagnosis system for pipeline robots was proposed based on sound signal recognition, which can provide a better understanding of the robot’s own situation without affecting the safe operation of the pipeline robot. This is a key issue to improve the reliability of the pipeline robot. The proposed fault prediction method achieved advanced results on the pipeline robot audio dataset and proved an effective application of the pretraining model in the field of pipeline robot fault diagnosis. Based on the idea of deep transfer learning, this model has the advantages of fast training speed and solves the problem of small datasets not being able to be trained in deep CNN. The results show that the system can monitor the pipeline robot’s own situation online, reducing the probability of serious failures of the pipeline robot, and can solve actual problems in engineering.

In practical applications, the limitations of the system are as follows: the sound recognition of the system did not include all fault cases, and better generalization performance should be achieved in the future. This method is expected to be applied to other pipeline robots in terms of fault diagnosis monitoring and therefore, it will continue to play an effective role in the field of fault detection and classification of different mechanical systems.

## Figures and Tables

**Table 1:** Signal-to-noise ratios of sound signals before and after denoising.

	Normal Signal	Motor Raceway Fault Signal	Crawler Wheel Stuck Fault Signal	Posture Adjustment Fault Signal
original sound signal	6.481	6.364	6.121	6.241
denoised signal	7.366	7.254	7.569	7.624

**Table 2:** Working condition of the pipeline robot.

	Condition	Description
NC	Normal condition	Healthy pipeline robot without defect
MC	Motor raceway fault	Motor raceway defect
MR	Motor roller fault	Motor roller defect
TJ	Crawler wheel stuck fault	The pipeline robot is stuck by a foreign object in the pipeline.
SA	Posture adjustment fault	Realize the adaptive fit between the track and the pipe wall under different pipe diameters through structural adjustment.
BN	Bearing fault	Bearing defect

**Table 3:** Classification results.

Fault Diagnosis Method	NC	MC	MR	TJ	SA	BN	Average
VGG16	96.99%	95.90%	95.93%	95.88%	95.91%	95.92%	95.99%
CNN trained from scratch	98.20%	97.55%	97.45%	98.20%	97.46%	96.46%	97.55%
Fine-tuned the last 1 residual block and SoftMax	97.88%	97.88%	97.34%	97.86%	97.88%	97.42%	97.71%
Only fine-tune the SoftMax	94.56%	93.46%	95.79%	93.73%	95.83%	96.03%	94.90%

## Supplementary Materials

The following are available online at https://www.mdpi.com/article/10.3390/s22093275/s1.
