# Supplementary figures and images for: A Fault Diagnosis System for a Pipeline Robot Based on Sound Signal Recognition
Source: Sensors (Basel). 2022 Apr 24;22(9):3275. doi: 10.3390/s22093275 (PMC9104926; doi:10.3390/s22093275)

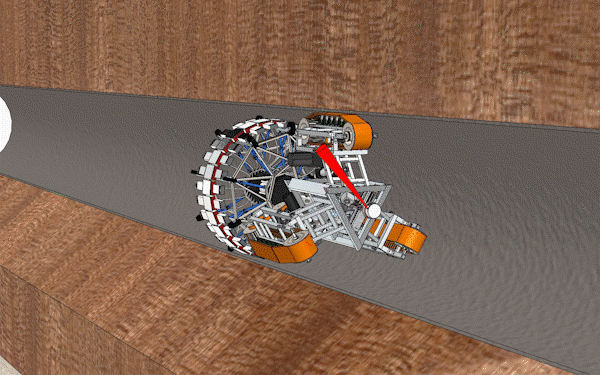

Supplement: Supplementary file 1 [file sensors-22-03275-s001.zip › image3.gif]
